# Spin Polarization Enhances the Catalytic Activity of Monolayer MoSe_2_ for Oxygen Reduction Reaction

**DOI:** 10.3390/molecules29143311

**Published:** 2024-07-13

**Authors:** Dan Shu, Dan Wang, Yan Wang, Liming Tang, Keqiu Chen

**Affiliations:** 1 School of Physics and Electronic Science, Hunan University of Science and Technology, Xiangtan 411201, China; shudan@mail.hnust.edu.cn; 2 Hunan Province Key Laboratory of Material Table Interface Science and Technology, School of Electronic Information and Physics, Central South University of Forestry and Technology, Changsha 410004, China; wangdan@hnu.edu.cn; 3 School of Information and Electrical Engineering, Hunan University of Science and Technology, Xiangtan 411201, China; 4 School of Physics and Electronics, Hunan University, Changsha 410082, China; lmtang@semi.ac.cn (L.T.); keqiuchen@hnu.edu.cn (K.C.)

**Keywords:** first–principles calculations, band unfolding, oxygen reduction reaction, valley splitting

## Abstract

The key factors in achieving high energy efficiency for proton exchange membrane fuel cells are reducing overpotential and increasing the oxygen reduction rate. Based on first-principles calculations, we induce H atom adsorption on 4 × 4 × 1 monolayer MoSe_2_ to induce spin polarization, thereby improving the catalytic performance. In the calculation of supercells, the band unfolding method is used to address the band folding effect in doped systems. Furthermore, it is evident from analyzing the unique energy band configuration of MoSe_2_ that a higher valley splitting value has better catalytic effects on the oxygen reduction reaction. We believe that the symmetries of the distinct adsorption site result in different overpotentials. In addition, when an even number of hydrogen atoms is adsorbed, the monolayer MoSe_2_ has no spin polarization. The spin can affect the electron transfer process and alter the hybrid energy with the reaction products, thereby regulating its catalytic performance.

## 1. Introduction

Proton exchange membrane fuel cells have high energy efficiency, small size, and reliable operation. However, the technology is still hindered by the slow kinetics and high overpotential of the oxygen reduction reaction (ORR) [1,2,3]. Therefore, it is necessary to develop efficient oxygen reduction catalysts to break the technical bottleneck [4]. As a significant member of the transition metal dichalcogenides (TMDCs) family, monolayer MoSe_2_ offers a promising opportunity for electrochemical energy systems because of its layered structure and the conductivity of Se atoms. However, the basal plane of TMDCs is usually inert [5,6,7]. Therefore, it is necessary to modify TMDCs in order to achieve the intended outcome. At present, most operating methods use Pt atoms instead of Mo atoms, thereby exhibiting excellent catalytic performance. However, this method has not been extensively utilized in commercial settings due to the high price of Pt [8,9]. Therefore, the reasonable design of non–precious metal electrode materials is the basis of the large–scale application of fuel cells.

Although the edge sites of two–dimensional TMDCs are highly active, the overall electrocatalytic performance of these TMDCs is limited due to the insufficient number of basal plane active sites [10,11]. Therefore, many techniques have been used to improve the catalytic performance, such as phase engineering, defect engineering, and structural engineering [12,13,14,15]. For example, Zhao and colleagues studied the characteristics of monolayer MoSe_2_ doped with various non-metal elements, including its geometry, electronic and electrical transport properties, oxygen reduction potential, and photocatalytic behavior. It has been discovered that adding non-metallic dopants with an odd number of valence electrons to MoSe_2_ can enhance its catalytic capabilities [16]. Meanwhile, transition metal atom doping has also been found to be an effective means of regulating the ORR rate. As a result, materials are modified to improve their transport properties and electronic structure [17,18]. The presence of Mo and Fe reduces the energy barrier of the proton coupling process, regulates the electronic structure, and improves the conductivity [19]. However, it is easy for the transition metal atoms to form clusters, which affects the stability of the battery. Recently, spin catalysis has attracted people’s attention. Spin is the fundamental property of the angular momentum of electrons and can have a unique effect on the electronic structure of materials [20,21]. Spin–injection and spin–orbit coupling (SOC) interactions cause electron spins to change along the reaction pathway [22]. Zhong et al. investigated the correlation between spin states and catalytic activity, but the underlying relationship is unclear [23]. The method of adjusting the spin state involves the application of magnetic fields. Li et al. reported that the moderate spin polarization of Fe results in excellent catalytic properties [24]. In addition, studies have shown that the high spin state energy of doped C is responsible for the adsorption of O_2_ at the doping site, and improves the ORR catalytic performance of h-BN [25]. In contrast to transition metal atoms, hydrogen has the simplest 1/2 spin, which can be well analyzed for spin effects. In fact, using hydrogen adsorption to regulate the electronic structure of two-dimensional materials is feasible and widely studied, and hydrogenation has been achieved in naturally occurring single–crystal TMDCs [26,27,28,29,30].

In this work, we anticipate employing a simple and intuitive approach to control the spin and magnetic moments in order to enhance the catalytic efficiency of monolayer MoSe_2_ through the absorption of spin-polarized atoms. Firstly, we use the method of band unfolding to solve the problem where the supercell doping system is not conducive to the analysis of impurity bands. Secondly, the electron structure of monolayer MoSe_2_ is analyzed by first-principles calculation. The findings indicate the presence of spin polarization upon adsorption of an odd quantity of H atoms. Spin polarization can break the symmetry of time inversion and realize valley splitting. Finally, we calculate the effect of spin polarization on catalysis. The results show that spin polarization successfully reduces the reaction overpotential and improves the catalytic efficiency. The internal mechanism of the catalyst is described from the aspects of structural stability, oxygen reduction overpotential, and electronic structure.

## 2. Results and Discussion

### 2.1. Geometric Structure

**Figure 1:**
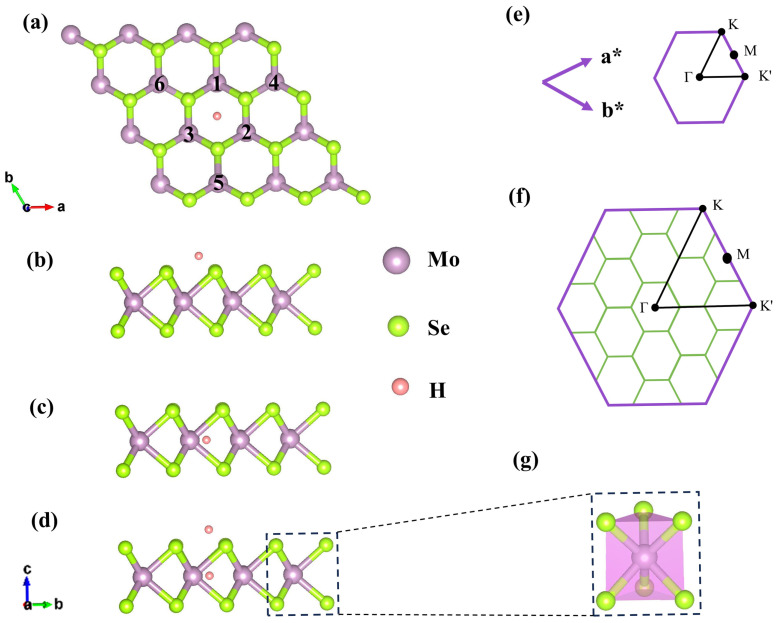
(**a**) Top view and (**b**–**d**) side view of the optimized monolayer MoSe_2_ adsorbing hydrogen atoms at A, C, and AC sites, respectively. (**e**,**f**) The first Brillouin zone and high symmetry points of the 1 × 1 × 1 primitive cell and 4 × 4 × 1 supercell, respectively. (**g**) The structure for trigonal prisms of monolayer MoSe_2_.

The monolayer MoSe_2_ features a binary honeycomb lattice where each molybdenum atom is positioned between six selenium atoms in a trigonal prismatic structure (as depicted in Figure 1g). We optimize the monolayer MoSe_2_ structure, and the relaxed lattice parameter after convergence is a = b = 3.32 Å, the Mo-Se bond length is 2.54 Å, and the Mo–Se–Mo bond angle is 81.56°, which is consistent with the result of 3.29 Å given in previous reports [31]. The stability of hydrogen atom adsorption sites is examined on 4 × 4 × 1 monolayer MoSe_2_. Results indicate that the system is most stable when hydrogen atoms are adsorbed at the center of the hexagonal ring on the anionic surface (referred to as the A position, Figure 1b) or the top of the center of the hexagonal ring on the cationic surface (referred to as the C position, Figure 1c), with adsorption energies of −0.28 eV and −0.91 eV, respectively. Figure 1d illustrates the adsorption of one hydrogen atom in the anion and one in the cation center. Following optimization, the lattice constants of sites A, C, and AC are 13.26 Å, 13.29 Å, and 13.27 Å, respectively, which are not significantly different from the original pure monolayer MoSe_2_ lattice constant a = 13.26 Å. Figure 1a provides a top view of a monolayer MoSe_2_ supercell adsorbing hydrogen atoms at different sites. The first Brillouin band of monolayer MoSe_2_ protocells and monolayer 4 × 4 × 1 MoSe_2_ supercells is shown in detail in Figure 1e,f, respectively, where we have labeled points of high symmetry. When the area of the first Brillouin band of the MoSe_2_ supercell becomes 1/16 of that of the original monomer (larger periods in real space correspond to smaller Brillouin band areas), the energy band of the supercell folds, and the high symmetry point of the original monomer is folded into the high symmetry point of the supercell [32].

### 2.2. Electronic Structure

**Figure 2:**
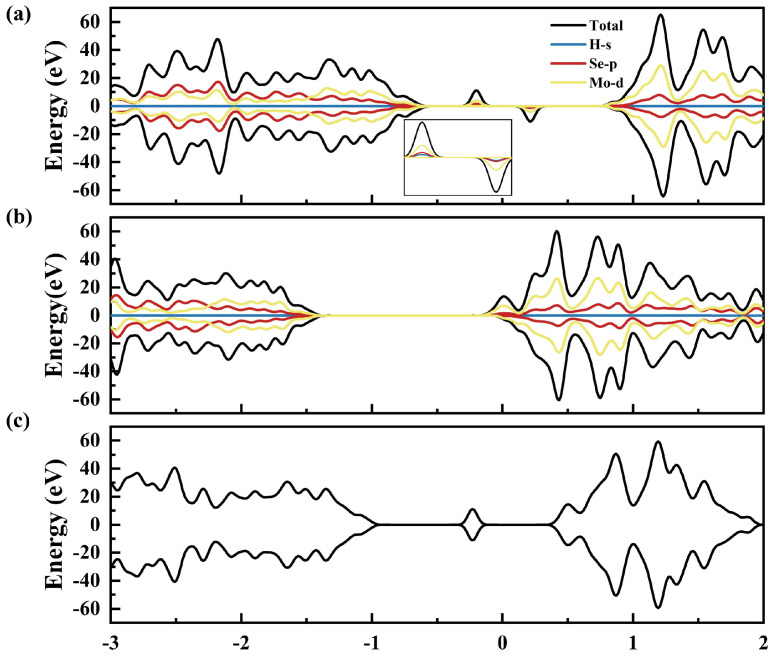
(**a**–**c**) The corresponding TDOS and PDOS for the hydrogen adsorbing at A, C, and AC, respectively.

To confirm the successful introduction of spin polarization, we calculate the total state density (TDOS) and partial state density (PDOS) of hydrogen adsorbed by A, C, and AC. Figure 2a reveals a coupling between H and MoSe_2_ when single H is adsorbed at the A site, introducing a localized spike near the Fermi level. For single H adsorption at the C site, the electronic structure changes, allowing the conduction band to pass through the Fermi level, as shown in the PDOS in Figure 2b. Notably, the states of spin-up and spin-down of monolayer MoSe_2_ are unequal, especially near the Fermi level in both A and C sites, confirming the injection of spin polarization into the MoSe_2_ layer. However, the AC site is completely symmetric, indicating no spin injection (Figure 2c).

**Figure 3:**
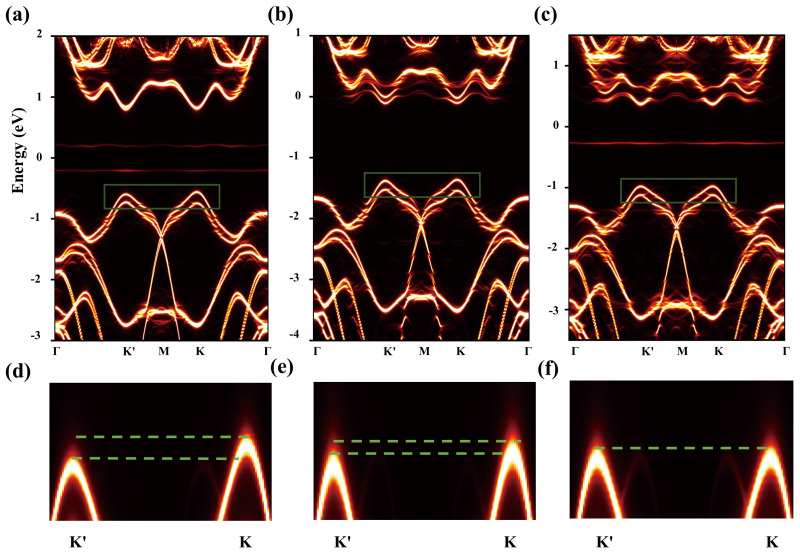
In the presence of SOC, monolayer MoSe_2_ supercells adsorbed the electron band structure of H at (**a**) A sites, (**b**) C sites, and (**c**) AC sites. (**d**–**f**) The corresponding enlarged details of the band structure. The red lines indicate the bands of the MoSe_2_ supercell. The green wire frame indicates the part of the energy valley to be enlarged.

In order to expand the band structure of the supercell, we use the technique of expanding the electron band to calculate the local k projection band. In Figure 3a–c, the band structures of adsorbed hydrogen atoms at the A, C, and AC sites of monolayer MoSe_2_ supercells are shown, as calculated in the presence of SOC. We find that spin splitting occurs in all three configurations, especially at the top of the valence band. Valley splitting is observed in both valence and conduction bands at positions A and C. A diagram of the local band structure of the valence band at the top of monolayer MoSe_2_ is enlarged in Figure 3d–f to better show the change of valley splitting value. It is noteworthy that the valley splitting value at the A site is greater than that at the C site, and no valley splitting occurs at the AC site when two hydrogen adsorbs are present. We know that the size of valley splits is usually defined as the difference between the energy extremes of the K valley and K’ valley; therefore, the expression of the valley splits of the valence band and conduction band is $\Delta_{\mathrm{val}}^{\mathrm{VB}(\mathrm{CB})}=E_{K}^{\mathrm{VB}(\mathrm{CB})}-E_{K^{\prime }}^{\mathrm{VB}(\mathrm{CB})}$, and the expression of total valley splits of TMDCs is $\Delta_{K,K^{\prime }}=\Delta_{\mathrm{VB}}-\Delta_{\mathrm{CB}}$. According to this formula, we calculate the valley splitting values at the valence band top and the conduction band bottom of A and C sites, which are 35 meV and 25 meV at the A site and −20 meV and 10 meV at the C site. Thus, the total valley splitting values are 55 meV and 15 meV for A and C, respectively, as shown in Table 1. The results show that the time inversion symmetry of monolayer MoSe_2_ is destroyed by injecting a spin-polarized H atom, thus achieving valley splitting.

**Figure 4:**
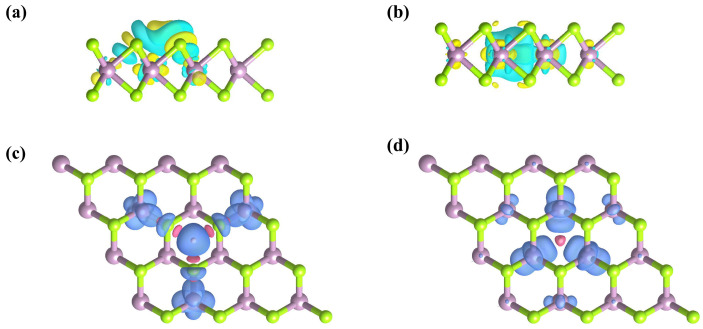
(**a**,**b**) Side view of the three-dimensional charge density difference plots for 4 × 4 × 1 monolayer MoSe_2_ adsorbing H atom at the A and C positions, respectively. The yellow and green indicate the charge increase and charge reduction, respectively. (**c**,**d**) Structure and spin density distribution of monolayer MoSe_2_ at A and C sites. The blue and red indicate the spin-up and spin-down states, respectively.

We calculate the charge density difference (CDD). As we can see, electrons accumulate on the H atom (yellow) and dissipate on the monolayer MoSe_2_ (green). It is evident from Figure 4a,b that the addition of spin-polarized hydrogen atoms results in charge transfer. Bader charge calculation shows that the A and C sites transfer to 0.51 e and 0.71 e, respectively. The transfer of electrons from MoSe_2_ to the hydrogen atom results in a redistribution of charge, creating an asymmetric electric field within the monolayer of MoSe_2_. In addition, we calculate the spin density distribution of monolayer MoSe_2_ at positions A and C. According to Figure 4c,d, the total magnetic moment at site A of the system is mainly provided by the Mo atoms numbered 4, 5, and 6, while the total magnetic moment at site C of the system is mainly provided by the Mo atoms numbered 1, 2, and 3 (Figure 1a). The results show that the magnetic moment of 0.76 μB/0.71 μB is generated by the adsorption of a hydrogen atom at the A or C site of 4 × 4 × 1 MoSe_2_. The H atom adsorbed at the A site is spin–up, while that at the C site is spin–down. Different spin states are indicated in red and blue, respectively (in Figure 4c,d). According to the Pauli exclusion principle, it is observed that there is no spin polarization at the AC site due to the fact that two electrons occupy the same energy level, leading to spin degeneracy. Thus, the spin-polarized impurity band will cause the carrier to undergo opposite energy changes at the K/K′ unequal valley, where one energy increases, and the other energy decreases. By regulating the electron transfer process, the hybridization energy with the reaction products can be changed, and, finally, the catalytic performance can be regulated.

### 2.3. Oxygen Reduction Reaction Analysis

**Figure 5:**
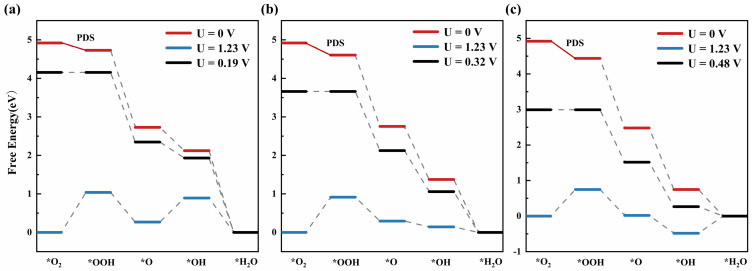
(**a**–**c**) These are the ORR free energy graphs of unadsorbed H atoms at the C site and A site at different potentials of monolayer MoSe_2_. Solid red lines indicate PDS. Ȍ*ȍ indicates adsorption.

The adsorption strength of O-containing intermediates (*O, *OH, and *OOH) will directly affect the electrochemical activity of the ORR. In order to understand the effect of spin polarization on the adsorption strength of intermediates, we obtain the free energy diagram by calculating the four-electron path of the ORR process. The potential determination step (PDS) is marked with a solid red line, as shown in Figure 5, and the reaction overpotential ($\eta_{\mathrm{ORR}}$) is calculated by Equation (2). The adsorption of O-containing intermediates is too strong and can easily hinder the reaction, while adsorption that is too weak cannot react. It can be seen from Figure 5 that the conversion of O_2_ to *OOH is the PDS of the reaction. When a single H atom is adsorbed by a single layer of MoSe_2_, the adsorption free energy of *OOH by this structure gradually decreases. In terms of catalytic efficiency, the ORR steps with high catalytic activity are mostly exothermic processes. Under the condition of equilibrium potential (1.23 V), the ORR step does not have a complete free energy decline trend, which indicates that ORR does not have the characteristics of spontaneous progress under this condition. As can be seen in Figure 5, the maximum allowable potentials of the A and C sites are 0.32 V and 0.44 V, respectively. However, for monolayer MoSe_2_ without adsorption of H atoms, the maximum potential allowed is 0.19 V. Spin polarization can affect the adsorption strength of O-containing intermediates, which leads to the change in overpotential. When hydrogen is adsorbed at site A, it effectively decreases the overpotential of the reaction ($\eta_{\mathrm{ORR}}$ = 0.75 V), demonstrating a remarkable catalytic impact. The entire reaction is exothermic and occurs spontaneously. It is found that spin polarization can reduce the reaction overpotential and improve the catalytic activity of the ORR. The greater the valley splitting value exhibited, the better the catalytic effect. Spin polarization can vary the electron transfer process of monolayer MoSe_2_, then change the hybrid energy with the reaction products, and, finally, lead to its catalytic performance.

We further analyze the reason for the difference in overpotential between site A and site C. The symmetry analysis is summarized in Table 2. When a hydrogen atom is adsorbed at site A, the system is C_3*v*_ symmetric. Where s orbitals belong to a_1_ representation, p orbitals and d orbitals belong to a_1_ e representation. Therefore, the s-p, s-d, and p-d orbitals allow coupling because they now have a common representation (a_1_). In the magnification of PDOS local spikes in Figure 2a, it can be seen that the s orbitals of H, the p orbitals of Se, and the d orbitals of Mo are coupled. In addition, the distance between the H atom and Se atom is closer at 2.09 Å, as shown in Table 1. The calculation results show that the s orbital energy of the H atom is similar to the p orbital energy of the Se atom in energy, being −6.49 eV and −6.51 eV, respectively. Therefore, there is a strong s–p coupling. For site C, the system is D_3*h*_ symmetric. Only s–d and p–d orbits are allowed to be coupled. It is a common irreducible representation of s and d orbitals (a′_1_). The p and d orbitals have a common irreducible representation (e′). The H atom is closer to the Mo atom at 1.95 Å (in Table 1). However, the d orbital energy of the Mo atom is −3.75 eV, and the energy gap between the d orbital and the s orbital of the H atom is large. So, the coupling of s–d orbitals is not easy. Although p–d coupling is also allowed in these systems, it is not taken into account since both the p and d orbitals are fully occupied. Therefore, the valley splitting value at the A site is higher than that at site C, while the overpotential at site A of 0.75 V is lower than that at site C (0.91 V). So, the A site is better for the catalytic effect than the C site.

## 3. Computational Methods

We utilize the first-principles calculation software Vienna ab initio simulation package (VASP. 5.4.4) based on density functional theory (DFT) [33,34]. The generalized gradient approximation (GGA) of the Perdew–Burke–Ernzerhof (PBE) exchange-correlation functional is employed [33]. The projector augmented wave (PAW) potential is used [35]. The cutoff energy is 400 eV, and the standard of force convergence is less than 0.02 eV. The structures do not stop optimization until the difference between the two total energies is less than 1 × 10^−5^ eV. The k-point sampling grid is generated by using Gamma, and the generated sampling k-point meshes are 3 × 3 × 1. A 20 Å vacuum layer is used to reduce the interaction between MoSe_2_ layers. In addition, we use the layered k-projection and unfolding electronic bands method to solve the band folding effect of primeval supercells [36]. VASPKIT is used for image processing analysis of some results [37]. To evaluate the stability of the system, the binding energy is calculated as follows:

(1) $$E_{b}=E_{\mathrm{Total}\;}-E_{{\mathrm{MoSe}}_{2}\;}-E_{H}$$

where $E_{\mathrm{Total}}$ and $E_{{\mathrm{MoSe}}_{2}}$ are the total energy of the system and the base, respectively. $E_{H}$ is the energy of the H atom. The formula for calculating overpotential is as follows [38]:

(2) $$\eta_{\mathrm{ORR}}=\max\left(\Delta G_{1},\Delta G_{2},\Delta G_{3},\Delta G_{4}\right)/e+1.23$$

## 4. Conclusions

In summary, we have described in detail the effects of spin polarization on the catalytic performance of monolayer MoSe_2_ by first-principles calculations. The impact of hydrogen atoms on the electronic configuration of a supercell through the band unfolding has been clearly revealed. When an odd number of hydrogen atoms is adsorbed, spin momentum will occur in the system, which leads to the emergence of valley splitting. In our calculation, the maximum valley splitting value reached 55 meV (A site), which is equivalent to the Zeeman splitting that can be caused at an external magnetic field of 275 T. In addition, site A was the optimal catalyst, with an overpotential of 0.75 V. The findings indicate that spin polarization can decrease the overpotential in a doped system, resulting in enhanced catalytic performance compared to undoped MoSe_2_ (1.04 V). Finally, it was found that higher valley splitting makes the catalytic performance better. We analyzed it from the symmetry point of view, where the s–p, s–d, and p–d orbital coupling was allowed in the C_3*v*_ symmetry. According to the results of the atomic distance and energy level difference between the orbits of the monolayer MoSe_2_, it was found that there was a strong s-p orbital coupling at the A site.

## Figures and Tables

**Table 1:** The valley splitting values of MoSe_2_ adsorbing the hydrogen atoms at different sites. The $d_{H–\;\;\mathrm{Mo}}$(Å)($d_{H-\;\;\mathrm{Se}}$(Å)) shows the distance between the hydrogen atom and the nearest Mo (Se) atoms of the hexagon ring. The $\Delta_{\mathrm{VB}}$ (meV), $\Delta_{\mathrm{CB}}$ (meV), and $\Delta_{K,K^{\prime }}$ (meV) donate the valence band, conduction band, and total valley splitting of the monolayer MoSe_2_, respectively.

Site	$d_{H–\;\;\mathrm{Mo}}$ (Å)	$d_{H-\;\;\mathrm{Se}}$ (Å)	$\Delta_{\mathrm{VB}}$ (meV)	$\Delta_{\mathrm{CB}}$ (meV)	$\Delta_{K,K^{\prime }}$ (meV)
A	3.26	2.09	35	−20	55
C	1.95	2.57	25	10	15
AC	–	–	–	0	0

**Table 2:** Irreducible representations of s, p, and d orbitals in the C_3*v*_ and D_3*h*_ point groups.

Site	Point Group	s	p	d
A	C_3*v*_	a_1_	a_1_⊕e	a_1_⊕e
C	D_3*h*_	a′_1_	a″_2_⊕e′	a′_1_⊕e′⊕e″

## Data Availability

The data presented in this study are available in the article and Supplementary Materials.

## Supplementary Materials

The following supporting information can be downloaded at: https://www.mdpi.com/article/10.3390/molecules29143311/s1.
