# Supplementary material for: Spin Polarization Enhances the Catalytic Activity of Monolayer MoSe2 for Oxygen Reduction Reaction
Source: Molecules. 2024 Jul 13;29(14):3311. doi: 10.3390/molecules29143311 (PMC11279673; doi:10.3390/molecules29143311)
Supplement: Supplementary file 1 [file molecules-29-03311-s001.zip › molecules-3094394-supplementary.pdf]

Spin polarization enhances the catalytic activity of monolayer $MoSe_2$ for oxygen reduction reaction

Dan Shu [1], Dan Wang [2], Yan Wang [3, *], Li-Ming Tang [4] and Ke-Qiu Chen [4]

1 School of Physics and Electronic Science, Hunan University of Science and Technology, Xiangtan 411201, China; shudan@mail.hnust.edu.cn

2 Hunan Province Key Laboratory of Material Table Interface Science and Technology, School of Electronic Information and Physics, Central South University of Forestry and Technology, Changsha 410004, China; wangdan@hnu.edu.cn

3 School of Information and Electrical Engineering, Hunan University of Science and Technology, Xiangtan 411201, China

4 School of Physics and Electronics, Hunan University, Changsha 410082, China * Correspondence: ywang8@hnust.edu.cn(Y.W.)

In electrochemistry, the basic reaction steps of four electrons under acidic conditions can be expressed as follows:

$$* + O_2 + H^+ + e^- \rightarrow * OOH \quad (S1)$$

$$* OOH + H^+ + e^- \rightarrow * O + H_2O \quad (S2)$$

$$* O + H^+ + e^- \rightarrow * OH \quad (S3)$$

$$* OH + H^+ + e^- \rightarrow H_2O \quad (S4)$$

Where * represents the active site of O containing intermediates (* O, * OH and * OOH) on the material surface. The Gibbs free energy of O-containing intermediates involved in ORR is calculated as follows:

$$\Delta G_{*O_xH_y} = G_{*O_xH_y-M} - G_M - \left[xG_{H_2O} - (2x-y)\, G_{H_2}/2\right] \quad (S5)$$

Where $G_{*O_xH_y-M}$, G_M, G_{H_2O} and G_{H_2} represent the total Gibbs free energy, the Gibbs free energy of the substrate, the Gibbs free energy of H_2O and the Gibbs free energy of H_2. The change in Gibbs free energy of the ORR step can be calculated as:

$$\Delta G_1 = \Delta G_{*OOH} - 4.92 \quad (S6)$$

$$\Delta G_2 = \Delta G_{*O} - \Delta G_{*OOH} \quad (S7)$$

$$\Delta G_3 = \Delta G_{*OH} - \Delta G_{*O} \quad (S8)$$

$$\Delta G_4 = -\Delta G_{*OH} \quad (S9)$$

Table S1. The Charge transfer (e) and magnetic moment for H atoms adsorbed in monolayer $MoSe_2$ supercells for various sites. Numbers are the sequence number of Mo atoms near the H atom.

**Site**	**Charge transfer (e)**	**Magnetic moment**	
		**Numbers**	μ_B
		Mo-4	0.19
**A**	0.51	Mo-5	0.19
		Mo-6	0.19
		Mo-1	0.19
**C**	0.71	Mo-2	0.15
		Mo-3	0.19
**AC**	1.73		

Table S2. Overpotential of materials with different doping.

	$MoSe_2$	A- $MoSe_2$	C- $MoSe_2$
η_{ORR} (V)	1.04	0.75	0.91

Table S3. The adsorption energy of different materials for O_2.

	$MoSe_2$	A- $MoSe_2$	C- $MoSe_2$
E_{ads} (eV)	-0.48	-0.72	-0.55
